# Silencing RNA-Mediated Knockdown of IFITM3 Enhances Senecavirus A Replication

**DOI:** 10.3390/pathogens13040290

**Published:** 2024-03-29

**Authors:** Shamiq Aftab, Eric Nelson, Michael Hildreth, Xiuqing Wang

**Affiliations:** 1 Department of Biology and Microbiology, South Dakota State University, Brookings, SD 57007, USA; shamiq.aftab@sdstate.edu (S.A.); michael.hildreth@sdstate.edu (M.H.); 2 Department of Veterinary and Biomedical Sciences, South Dakota State University, Brookings, SD 57007, USA; eric.nelson@sdstate.edu

**Keywords:** interferon-induced transmembrane protein 3 (IFITM3), Senecavirus A (SVA), autophagy

## Abstract

Senecavirus A (SVA) is a non-enveloped, positive sense, single-stranded RNA virus that causes vesicular diseases in pigs. Interferon-induced transmembrane 3 (IFITM3) is an interferon-stimulated gene (ISG) that exhibits broad antiviral activity. We investigated the role of IFITM3 in SVA replication. Both viral protein expression and supernatant virus titer were significantly increased when endogenous IFITM3 was knocked down by approximately 80% in human non-smallcell lung carcinoma cell line (NCI-H1299) compared to silencing RNA control. Interestingly, overexpression of exogenous IFITM3 in NCI-H1299 cells also significantly enhanced viral protein expression and virus titer compared to vector control, which was positively correlated with induction of autophagy mediated by IFITM3 overexpression. Overall, our results indicate an antiviral role of endogenous IFITM3 against SVA. The exact molecular mechanisms by which endogenous IFITM3 limits SVA replication remain to be determined in future studies.

## 1. Introduction

Host cells detect viral invasion by recognizing pathogen-associated molecular patterns (PAMPs) and induce the production of type I interferons (IFNs) [1]. IFNs, in turn, trigger the production of IFN-stimulated genes (ISGs), which exhibit direct antiviral activities and restrict virus replication [1]. Interferon-inducible transmembrane proteins (IFITMs), a family of small transmembrane proteins, are basally expressed in a variety of cell types and potently induced by IFNs [1,2,3]. There are five known IFITM genes in humans. IFITM1, 2, and 3 restrict cytosolic entry of viral genomes by inhibiting direct fusion of viral cell membrane of enveloped viruses [4].

IFITM3 is known to restrict the replication of a broad range of enveloped viruses that enter cells by endocytosis, such as West Nile virus (WNV), dengue virus, influenza A virus (IAV), human metapneumovirus, and severe acute respiratory syndrome coronavirus (SARS-CoV-2) etc. [5,6,7,8,9,10,11]. IFITM3 is recruited to endosomes during early IAV infection [12] and inhibits virus replication by interfering with viral cell membrane fusion and cytosolic entry of viral genomes [6,13,14]. Similar mechanisms have been described for other enveloped RNA viruses [5,7,10]. IFITM3 has also been reported to restrict production of inflammatory cytokines induced by cytomegalovirus, IAV, and SARS-CoV-2 [15]. The role of IFITM3 in protecting mice from lethal challenges with foot-and-mouth disease virus (FMDV) and IAV-associated heart tissue damages has been reported [16,17].

Autophagy is an intracellular process that involves removing large amounts of damaged cytoplasmic proteins and organelles in a double-membrane vesicle called autophagosomes, which are shuttled to lysosomes for their degradation [18]. When autophagy is activated, microtubule-associated protein light chain 3-I (MAP-LC3-I) is converted into a lipidated form, LC3-II, which is membrane-bound [19]. An increased expression of LC3-II and/or increased LC3-II/LC3-I ratio are often used as markers of autophagy [20]. Many virus infections, such as poliovirus, coxsackie virus B3, IAV, dengue virus, hepatitis C virus, and Senecavirus A (SVA), have been shown to induce autophagy and exploit autophagy for their replication [19,21,22,23,24,25,26,27,28]. Autophagy can also exhibit antiviral activity and acts as a double-edged sword during virus infection [29]. Overexpression of IFITM3 and other ISGs, such as interferon-induced guanylate-binding protein 1 (GBP1), have been reported to induce autophagy [30,31,32].

Senecavirus A (SVA), also known as Seneca Valley Virus (SVV), belongs to genus *Senecavirus* of the *Picornaviridae family* [33]. SVA is a non-enveloped, positive-sense, single-stranded RNA virus that causes vesicular diseases in sows and neonatal piglets [34]. The clinical signs of SVA infection are similar to those of other swine viral diseases, including foot-and-mouth disease [35]. Persistent SVA infection and possible transmission of SVA from carrier sows to contact piglets have been reported [36]. Furthermore, SVA can be potentially transmitted to susceptible animals through contaminated feed [37].

SVA binds to anthrax toxin receptor 1 (ANTXR1) and enters host cells via the cholesterol-mediated endocytic pathway [38]. Other studies have suggested that SVA is internalized via multiple endocytic pathways, including clathrin heavy chain (CLTC)-mediated endocytosis (CME), caveola/lipid-raft-mediated endocytosis, and cholesterol-mediated endocytosis [39]. Upon internalization, SVA is transported to late endosomes and lysosomes in a pH-dependent manner [40]. The role of IFITM3 in restricting the replication of several non-enveloped RNA viruses by interfering with the disassembly of capsid and cytosolic entry of viral RNA genomes has been reported [16,41,42,43]. To our knowledge, there is no published report on the role of IFITM3 on SVA replication. A recent study by Xu et al. (2022) showed that IFN-induced protein with tetratricopeptide repeats 3 (IFIT3) inhibits SVA replication by interfering with viral entry, virus assembly, and release [44]. Here, we have, for the first time, provided evidence to show antiviral activity of endogenous IFITM3 against SVA. Overexpression of IFITM3 in NCI-H1299 cells induced autophagy and enhanced SVA replication.

## 2. Materials and Methods

### 2.1. Cell Cultures and Plasmid Transfection

NCI-H1299 cells (ATCC, CRL-5803) were cultured in Dulbecco’s modified essential medium (DMEM) supplemented with 10% fetal bovine serum (FBS) and 1% penicillin-streptomycin at 37 °C in a humidified 5% CO_2_ incubator. Cells were grown in 12-well plates at a seeding density of 1.2 × 10^5^ cells per well. After 24 h incubation, cells were transfected with 1 μg per well of pQCXIP control vector (Q-series retroviral vector; Retro-X Q Vector Set) (Takara Bio USA Inc. Mountain View, San Jose, CA, USA, Cat. No. 631516) or pQCXIP expressing IFITM3-HA (pQCXIP-IFITM3) [45] using Lipofectamine-3000 Reagent (Invitrogen, Carlsbad, CA, USA, Cat. No. L3000015) following the manufacturer’s protocol. At 48 h after transfection, cells were infected with SVA isolate SD15-26 [46] at a MOI of 0.1 for 24 h. Cells and supernatant were collected after 24 h post infection and stored at −80 °C for further analysis.

In some experiments, NCI-H1299 cells were cultured in a 12-well plate at 1.2 × 10^5^ cells per well. After 24 h incubation, cells in triplicates were pre-treated with 3-Methyl Adenine (3-MA) (Sigma Aldrich, Saint Louis, MO, USA, Cat. No. M9281) in DMSO at a final concentration of 5 mM for 4 h. Three wells were marked as control (without 3-MA) and incubated with equal amount of DMSO in culture media. Cells were then infected with SVA isolate SD15-26 at a MOI of 0.1 for 24 h. Supernatant was collected, and viral titer was determined by TCID_50_ assay using the Spearman–Karber method [47,48].

### 2.2. siRNA-Mediated Knockdown of IFITM3 in H1299 Cells

NCI-H1299 cells were cultured in 12-well plates at a seeding density of 1.2 × 10^5^ cells per well. After 24 h incubation, cells were transfected with either negative control siRNA (Ambion, Austin, TX, USA, Cat. No. AM4642) or 60 nM of IFITM3 siRNA (Life Technologies, Carlsbad, CA, USA, siRNA ID# s195035) using Lipofectamine RNAiMAX Transfection Reagent (Invitrogen, Carlsbad, CA, USA, Cat. No. 13778075) following manufacturer’s protocol. After 48 h post transfection, each well was infected with SVA isolate SD15-26 at a MOI of 0.1 for 24 h. Cells and supernatant were collected and stored at −80 °C for further analysis.

### 2.3. Quantitative Reverse Transcription PCR (qRT-PCR)

Total RNA was extracted from cells by using RNeasy Mini Kit (Qiagen, North Rhine-Westfalia, Germany, Cat. No. 74104), in accordance with the manufacturer’s instructions. High-Capacity cDNA Reverse Transcription Kit (Applied Biosystems, Foster City, CA, USA, Cat. No. 4368814) was used to make cDNA from 1 µg of total RNA by following the manufacturer’s instructions. qPCR was performed with Brilliant II SYBR Green qPCR (Agilent Technologies, Santa Clara, CA, USA, Cat. No. 600828) and three µL of cDNA was used in each qPCR reaction. The following forty cycles of PCR condition were used: 95 °C for 30 s, 55 °C for 30 s, and 72 °C for 30 s. Primers specific to beta-actin (FP: TTGCTGACAGGATGCAGAAGGAGA; RP: ACTCCTGCTTGCTGATCCACATCT), IFITM3 (FP: GGTCTTCGCTGGACACCAT; RP: TGTCCCTAGACTTCACGGAGTA), and SVA-VP2 (FP: TCCTGTAAGCTCTTTGCCTTG; RP: TCTTAATGAAGTGCGGGTGG) were used. The reactions were run in a QuantStudio 6 Flex Real-Time PCR System (Applied Biosystems). The ΔΔCT method was utilized to calculate fold change in gene expression, as described previously [49].

### 2.4. Western Blot Analysis

Cells were harvested and lysed using cell lysis buffer (0.01 M Tris-HCl pH 8, 0.14 M NaCl, 0.025% NaN_3_, 1% Triton X-100) supplemented with 1:100 Halt^TM^ protease and phosphatase inhibitor cocktail (ThermoScientific, Rockford, IL, USA, Cat. No. 78441) for 15 min at 4 °C, alternating with vortexing for 15 s, repeated 4 times. Cell lysates were centrifuged at 13,000× *g* for 5 min at 4 °C and supernatants were collected. Equal amounts of cell lysates were loaded onto a 4–20% Tris-Glycine gel (Invitrogen, Carlsbad, CA, USA, Cat. No. XP04205BOX) and separated proteins were subsequently transferred onto nitrocellulose membrane (Amersham, Marlborough, MA, USA, Cat. No. 10600048). The membranes were then blocked in 5% non-fat dry milk for 1 h at room temperature and incubated with primary antibody prepared in blocking buffer at 4 °C overnight with gentle shaking. The following primary antibodies were used: mouse monoclonal anti-HA antibody (Sigma-Aldrich, St. Louis, MO, USA, Cat. No. H9658) at 1:5000, mouse monoclonal anti-beta-actin antibody (Sigma-Aldrich, St. Louis, MO, USA, Cat. No. A2228) at 1:5000, rabbit anti-IFITM3 polyclonal antibody (Proteintech, Rosemont, IL, USA, Cat. No. 11714-1-AP) at 1:1000 dilution, rabbit anti-LC3-II antibody (Sigma-Aldrich, St. Louis, MO, USA, Cat. No. L7543) at 1:2000, mouse monoclonal anti-VP2 (SVA capsid protein) antibody at a dilution of 1:500 [50]. After washing 3 times for 5 min with PBS containing 0.1% Tween (Sigma-Aldrich, St. Louis, MO, USA, Cat. No. P1379), membranes were incubated in the dark with goat anti-mouse IRDye^®^ 800 CW conjugated 2° Ab (LI-COR, Lincoln, NE, USA, Cat. No. 926-32210) or donkey anti-rabbit IRDye^®^ 680RD conjugated 2° Ab (LI-COR, Lincoln, NE, USA, Cat. No. 926-68073) at 1:15,000 dilution for 1 h at room temperature. Membranes were washed 3 times for 5 min in PBST. Images were captured using a LI-COR Odyssey Infrared Imaging System. Quantitative analysis of Western blot image was performed using Fiji software (https://imagej.net/software/fiji/downloads accessed on 23 March 2024) (ImageJ2, Madison, WI, USA) [51]. Beta actin was used as a loading control to normalize protein expression level.

### 2.5. Confocal Immunofluorescence Analysis

For confocal microscopy, NCI-H1299 cells were cultured in 8-well chamber slides (ThermoScientific, Rochester, NY, USA, Cat. No. 177402) at a seeding density of 30,000 cells per well. Following 24 h incubation, well 1 and 2 were co-transfected with 200 ng of EGFP-LC3, along with either 200 ng of pQCXIP vector control or pQCXIP-IFITM3, and well 3 and 4 were transfected with 400 ng per well of EGFP-LC3 using Lipofectamine-3000 reagent following the manufacturer’s protocol. Cells transfected with EGFP-LC3 alone were treated with Torin1 (Cell Signaling, Danvers, MA, USA, Cat. No. 14379) at a final concentration of 500 nM (1:2000) for 3 h. EGFP-LC3-transfected cells were also infected with SVA isolate SD15-26 at a MOI of 0.1 for 24 h. Both Torin1-treated and SVA isolate SD15-26-infected cells were considered as controls. Cells were then fixed with 1% paraformaldehyde for 10 min and washed with PBS for 5 min. Cells were permeabilized using 0.1% Triton X-100 for 5 min at room temperature and washed again with PBS. The cells were blocked with 5% goat serum in PBS for 30 min at room temperature and then incubated with anti-LAMP-1 (1D4B) (SCBT, Santa Cruz, Dallas, TX, USA, Cat. No. sc-19992) conjugated to Alexa 647 (1:50) at room temperature for 2 h. The cells were then washed three times with PBS and counter-stained with 4′,6-Diamidino-2-phenylindole (DAPI) to stain the nucleus for 2 min and mounted using ProLong Gold antifade mounting reagent (Invitrogen, Eugene, OR, USA, Cat. No. P36930). Slides were observed under Olympus Fluoview FV1200 Laser Scanning Confocal Microscope and images were taken at 40X magnification. The number of DAPI-stained cells exhibiting yellow fluorescence, which is caused by an overlap of the emission spectra for green (EGFP) and red (Alexa 647), were counted in colocalization analysis. The mean percentage of colocalization was calculated by randomly choosing five cells for every condition, and the threshold range was then chosen using manual thresholding to distinguish the object of interest from the background using Fiji default thresholding method [51]. The area of the colocalized region (X) was measured by adjusting the color (yellow) threshold. The area of all five cells was then obtained by adjusting the threshold color to pick all five cells (Y). The fraction (X/Y) was multiplied by 100 to determine the percentage colocalization. For every condition, mean percentage of colocalization was determined from five cells in three randomly chosen areas.

### 2.6. Statistical Analysis

Statistical analysis was conducted using GraphPad Prism version 10.1.2 (GraphPad software, San Diego, CA, USA). A two-tailed unpaired Student’s *t*-test was used to compare the SVA titer between pQCXIP vector control and IFITM3-HA-transfected NCI-H1299 cells. A one-way analysis of variance (ANOVA) was used to determine the statistically significant differences for LC3-II/LC3-I and mean percentage of colocalization experiment. A Tukey’s test was used to determine the significant differences in LC3-II/LC3-I in IFITM3-overexpressed NCI-H1299 cells when compared to the vector control, and SVA isolate SD15-26-infected and Torin1-treated NCI-H1299 cells when compared to mock. For mean percentage of colocalization, Tukey’s test was used to determine the significant differences between mean percentage of pQCXIP’s empty vector when compared to pQCXIP-IFITM3, SVA, and Torin1. *p*-value < 0.05 was considered statistically significant.

## 3. Results

### 3.1. Knockdown of Endogenous IFITM3-Enhanced SVA Replication

**Figure 1:**
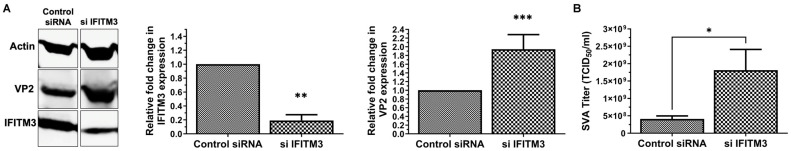
Knockdown of endogenous IFITM3 enhances replication of SVA in NCI-H1299 cells. (**A**): Western blot analysis showing expression of beta-actin, SVA VP2, and IFITM3 using mouse monoclonal antibodies specific for beta-actin and SVA VP2 and rabbit anti-IFITM3 antibody. A representative Western blot image and quantitative analysis of Western blot are shown. Graphs represent mean with standard deviations of three replicates. ** indicates *p* < 0.01; *** indicates *p* < 0.001. (**B**): Knockdown of IFITM3 increases supernatant virus titer. Graph represents the means with standard deviations of three replicates. * indicates *p* < 0.05.

To examine whether knockdown of endogenous IFITM3 affects SVA replication, NCI-H1299 cells were transfected with either siRNA specific for IFITM3 or control siRNA followed by SVA isolate SD15-26 infection at 48 h post transfection. Western blot analysis showed an average of 80% knockdown of endogenous IFITM3 compared to control siRNA (Figure 1A, middle panel, *p* < 0.01). IFITM3 silencing enhanced the expression of SVA capsid protein VP2 by an average of 1.94-fold at 24 h after SVA isolate SD15-26 infection (Figure 1A, right panel, *p* < 0.001). Virus titer in the supernatant of IFITM3-siRNA-transfected NCI-H1299 cells was increased by an average of 4.4-fold compared to negative control siRNA (Figure 1B, *p* < 0.05). Collectively, the data suggest an antiviral role of endogenous IFITM3 against SVA.

### 3.2. Overexpression of Exogenous IFITM3-Enhanced SVA Replication

**Figure 2:**
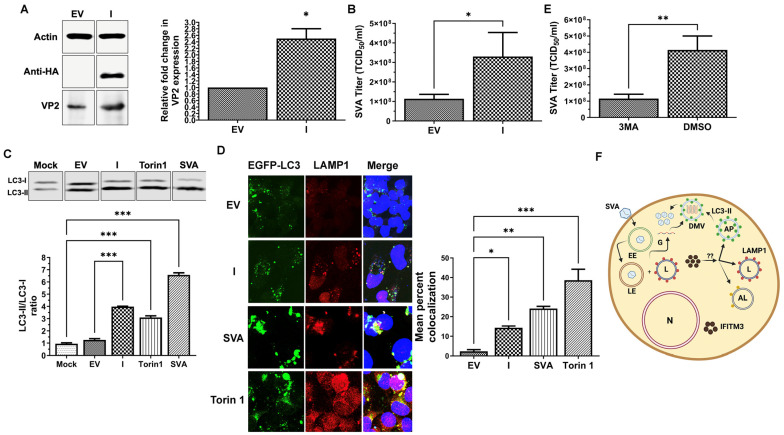
Overexpression of exogenous IFITM3 in NCI-H1299 cells enhances SVA replication and induces autophagy. (**A**): Representative Western blot showing expression of beta-actin, exogenous IFITM3, and SVA VP2 using mouse monoclonal antibodies specific for beta-actin, HA tag, and SVA VP2, respectively. EV: empty vector (pQCXIP). I: pQCXIP-IFITM3. Graph represents mean with standard deviations of three replicates. * indicates *p* < 0.05. (**B**): Overexpression of exogenous IFITM3 increases supernatant virus titer. Graph represents means with standard deviations of three replicates. * indicates *p* < 0.05. (**C**): Western blot analysis was performed to determine the expression of LC3-II and LC3-I. Graph represents means with standard deviations of three replicates. *** indicates *p* < 0.001. (**D**): Confocal microscopy image analysis showed colocalization of EGFP-LC3 with late endosome marker LAMP1. pQCXIP-IFITM3-transfected cells (I) compared to empty vector (EV). * indicates *p* < 0.05. ** indicates *p* < 0.01. *** indicates *p* < 0.001. (**E**): Autophagy facilitates SVA replication in NCI-H1299 cells. Graph represents means with standard deviations of three replicates. ** indicates *p* < 0.01. (**F**): A schematic diagram of our working hypothesis on the possible role of IFITM3-overexpression-mediated autophagy in enhancing SVA replication. N: nucleus. EE: early endosome. LE: late endosome. G: viral genome. L: lysosome. AP: autophagosome. AL: autophagolysosome. DMV: double-membraned vesicle.

To further confirm the antiviral role of IFITM3 in SVA replication, we employed the ectopic overexpression of exogenous IFITM3 approach. NCI-H1299 cells were either transfected with plasmid containing IFITM3-HA (pQCXIP-IFITM3) or empty vector pQCXIP. Western blot analysis showed the expression of HA-tagged IFITM3 in pQCXIP-IFITM3-transfected cells, but not in empty control-vector-transfected cells (Figure 2A). Surprisingly, an average of 2.5-fold increase in SVA VP2 expression was observed in IFITM3 overexpressing cells compared to pQCXIP at 24 h after SVA isolate SD15-26 infection (Figure 2A). A significant increase of supernatant virus titer was observed in IFITM3-overexpressing cells compared to pQCXIP (Figure 2B) (*p* < 0.05). Overall, overexpression of exogenous IFITM3 increased SVA replication.

### 3.3. Overexpression of IFITM3 in NCI-H1299 Induces Autophagy

Previous studies have shown that overexpression of IFITM3 induces autophagy in some cells [30,52]. To determine whether overexpression of exogenous IFITM3 induces autophagy in NCI-H1299 cells, we performed both Western blot analysis and confocal microscopy to assess the expression of LC3-II and colocalization of LC3 with late endosome and lysosomal marker LAMP1. We used LC3-II/LC3-I ratio as a marker of autophagy, as described previously [20]. As shown in Figure 2C, overexpression of exogenous IFITM3 showed a significant increase of LC3-II/LC3-I ratio when compared to the empty vector (*p* < 0.05). Torin1-treated NCI-H1299 cells were included as positive controls, which showed a significant increase of LC3-II/LC3-I ratio when compared to mock (Figure 2C) (*p* < 0.01). SVA isolate SD15-26 infection also caused a significant increase in LC3-II/LC3-I ratio when compared to mock (Figure 2C) (*p* < 0.001).

Co-localization of LC3 with late endosome/lysosome marker LAMP1 is typically considered a marker for autophagy [19]. To further confirm that overexpression of exogenous IFITM3 induces autophagy in NCI-H1299 cells, cells were co-transfected with either IFITM3-HA-expressing plasmid (I) or empty vector (EV) and EGFP-LC3. SVA isolate SD15-26 infected cells and Torin1-treated cells were included as controls. As shown in Figure 2D, an average of 14.3%, 24%, and 38.3% colocalization of EGFP-LC3 with LAMP1 was observed in IFITM3 overexpressing NCI-H1299 cells, SVA isolate SD15-26 infected NCI-H1299 cells, and Torin1-treated NCI-H1299 cells, respectively, while the EV group only showed 1.9% of colocalization between EGFP-LC3 and LAMP1. A significant increase in EGFP-LC3 and LAMP1 colocalization was observed in all groups compared to EV group. LAMP1 expression was upregulated in all groups compared to EV group. Both Western blot and confocal microscopy data support that exogenous IFITM3 overexpression induces autophagy in NCI-H1299 cells.

### 3.4. Autophagy Inhibition Reduces SVA Replication in NCI-H1299 Cells

Previous studies have shown that autophagy inhibits SVA replication in H1299 cells [26]. To verify that autophagy inhibition by 3-Methyl Adenine (3-MA) affects SVA replication, NCI-H1299 cells were treated with 3-MA and infected with SVA isolate SD15-26 at a MOI of 0.1 for 24 h. A significant reduction in virus titer was observed in 3-MA-treated NCI-H1299 cells compared to the DMSO-treated group (Figure 2E) (*p* < 0.05). The results suggest that inhibition of autophagy reduces SVA replication in NCI-H1299 cells.

## 4. Discussion

Most viruses enter host cells through either receptor-mediated endocytosis or viral cell plasma membrane fusion. Following virus entry by receptor-mediated endocytosis, uncoating by proteolytic disassembly of capsid coat of non-enveloped viruses or viral cell endosomal membrane fusion for enveloped viruses, which occur in late endosome/lysosome, allow cytosolic entry of viral genomes and replication of viral genomes and viral protein synthesis. IFITM3 is an ISG that localizes in late endosome/lysosome and modifies the fluidity of the membrane to restrict cytosolic entry of many viruses into host cells [6,10,13,42,52]. IFITM3 has no effect on viruses that enter cells through either viral cell plasma membrane fusion, such as Sendai virus [53,54] or reovirus infectious subvirion particles (ISVPs), which do not require endosomal proteolysis [41].

SVA enters cells by receptor-mediated endocytosis, followed by proteolytic cleavage of the capsid coat in late endosome/lysosomes and release of viral genomic RNA into cytosol [55]. Therefore, it is reasonable to speculate that endogenous IFITM3 also restricts SVA replication via cytosolic entry of viral RNA. We have indeed observed that endogenous IFITM3 exhibits an antiviral role against SVA replication (Figure 1). Furthermore, we have observed no difference in viral RNA copies between silencing RNA control and IFITM3 silencing RNA-transfected cells at 3 h post infection. While a significant increase in viral RNA copies was observed in IFITM3 silencing cells compared to control silencing cells at 8 h post infection (Supplementary Figure S1). Our data suggest that IFITM3 likely restricts SVA via cytosolic entry of viral RNA, but not through virus attachment and entry into the endosome. In contrast, one recent study has shown that IFITM3 also inhibits FMDV attachment and entry to cells, in addition to disassembly of the capsid in late endosomes [16]. Both FMDV and SVA belongs to the *Picornaviridae* family. Future studies are needed to better understand the molecular mechanisms by which endogenous IFITM3 restricts SVA replication.

Ectopic expression of exogenous IFITM3 is commonly used to assess the antiviral activities of IFITM3 in vitro [6,10,16,41]. Overexpression of IFITM3 has been consistently shown to restrict virus replication by affecting the fluidity of late endosomal membranes to prevent viral cell membrane fusion for enveloped viruses and disassembly of the capsid coat for non-enveloped viruses for the subsequent cytosolic entry of viruses, as described above. To our surprise, IFITM3 overexpression in NCI-H1299 cells enhanced SVA replication in vitro (Figure 2), despite the demonstrated antiviral activity of endogenous IFITM3 to SVA (Figure 1). Overexpression of IFITM3 has been reported to induce autophagy in some cancer cell lines. Our data further support this observation, as evidenced by the increased LC3-II expression and increased colocalization of EGFP-LC3 and LAMP1 (Figure 2C,D). Our data also demonstrate that SVA infection induces autophagy in NCI-H1299 cells.

Previous studies have reported that SVA-induced autophagy promoted SVA replication in PK-15 and BHK-21 cells, but inhibited SVA replication in NCI-H1299 and HEK-293T cells [25,26]. We have further explored whether inhibition of autophagy by 3-MA would enhance SVA replication in NCI-H1299 cells. Inhibition of autophagy by 3-MA has been shown to reduce replication of hepatitis B virus [56]. We observed that 3-MA significantly reduced SVA replication in NCI-H1299 cells (Figure 2E), suggesting autophagy is important in promoting SVA replication in NCI-H1299 cells. The positive correlation between autophagy induced by IFITM3 overexpression in NCI-H1299 cells and enhanced SVA replication we observed in this study provides further support to the hypothesis that SVA replication benefits from autophagy induction. Our working hypothesis is schematically shown in Figure 2F. Overexpression of exogenous IFITM3 triggers the induction of autophagy, as evidenced by increased LC3-II expression and co-localization of LC3 with LAMP1. It has been shown that double-membrane vesicles (DMV) or autophagosome facilitate the entry of FMDV [57]. The role of the autophagy pathway in facilitating FMDV replication has been described previously [58]. We speculate that autophagosome or DMV may also facilitate SVA replication since both FMDV and SVA belong to *Picornaviridae.* The detailed molecular mechanisms for the proposed working hypothesis remain to be determined. Furthermore, whether the enhanced SVA replication in IFITM3-overexpressing cells is caused by autophagy needs to be verified using autophagy defective stable NCI-H1299 cell lines in future studies. It has been reported that autophagy induced by IFITM3 overexpression does not contribute to its antiviral activity against IAV. Autophagy can be a double-edged sword during virus infection [29]. Some viruses, such as picornaviruses, have been shown to exploit autophagy for their replication through multiple mechanisms [29]. Autophagy can also restrict replication of other viruses, such as Sindbis virus (SINV), by degrading viral components through virophage [29]. More detailed studies are needed to better understand the dynamic nature of interaction between autophagy and SVA replication in different cell culture systems.

## 5. Conclusions

In conclusion, knockdown of endogenous IFITM3 enhanced SVA VP2 expression and virus titer. Overexpression of exogenous IFITM3 also enhanced SVA VP2 expression and virus titer, which is positively correlated with induction of autophagy by IFITM3 overexpression. The molecular mechanisms by which endogenous IFITM3 restricts SVA replication and the role of autophagy in enhancing SVA replication in IFITM3-overexpressing NCI-H1299 cells remain to be determined in future studies.

## Data Availability

No new data were created or analyzed in this study. Data sharing is not applicable to this article.

## Supplementary Materials

The following supporting information can be downloaded at: https://www.mdpi.com/article/10.3390/pathogens13040290/s1, Figure S1: IFITM3 restricts SVA replication post virus attachment and entry.

## References

[1] Schneider W.M., Chevillotte M.D., Rice C.M. (2014). Interferon-stimulated genes: A complex web of host defenses. Annu. Rev. Immunol..

[2] Diamond M.S., Farzan M. (2013). The broad-spectrum antiviral functions of IFIT and IFITM proteins. Nat. Rev. Immunol..

[3] Liao Y., Goraya M.U., Yuan X., Zhang B., Chiu S.H., Chen J.L. (2019). Functional Involvement of Interferon-Inducible Transmembrane Proteins in Antiviral Immunity. Front. Microbiol..

[4] Smith S.E., Weston S., Kellam P., Marsh M. (2014). IFITM proteins—Cellular inhibitors of viral entry. Curr. Opin. Virol..

[5] Jiang D., Weidner J.M., Qing M., Pan X.-B., Guo H., Xu C., Zhang X., Birk A., Chang J., Shi P.-Y. (2010). Identification of Five Interferon-Induced Cellular Proteins That Inhibit West Nile Virus and Dengue Virus Infections. J. Virol..

[6] Feeley E.M., Sims J.S., John S.P., Chin C.R., Pertel T., Chen L.M., Gaiha G.D., Ryan B.J., Donis R.O., Elledge S.J. (2011). IFITM3 inhibits influenza A virus infection by preventing cytosolic entry. PLoS Pathog..

[7] McMichael T.M., Zhang Y., Kenney A.D., Zhang L., Zani A., Lu M., Chemudupati M., Li J., Yount J.S. (2018). IFITM3 Restricts Human Metapneumovirus Infection. J. Infect. Dis..

[8] Xing H., Ye L., Fan J., Fu T., Li C., Zhang S., Ren L., Bai J. (2020). IFITMs of African Green Monkey Can Inhibit Replication of SFTSV but Not MNV In Vitro. Viral Immunol..

[9] Wang Z., Tuo X., Zhang J., Chai K., Tan J., Qiao W. (2022). Antiviral role of IFITM3 in prototype foamy virus infection. Virol. J..

[10] Zhang A., Duan H., Zhao H., Liao H., Du Y., Li L., Jiang D., Wan B., Wu Y., Ji P. (2020). Interferon-Induced Transmembrane Protein 3 Is a Virus-Associated Protein Which Suppresses Porcine Reproductive and Respiratory Syndrome Virus Replication by Blocking Viral Membrane Fusion. J. Virol..

[11] Xu F., Wang G., Zhao F., Huang Y., Fan Z., Mei S., Xie Y., Wei L., Hu Y., Wang C. (2022). IFITM3 Inhibits SARS-CoV-2 Infection and Is Associated with COVID-19 Susceptibility. Viruses.

[12] Kummer S., Avinoam O., Krausslich H.G. (2019). IFITM3 Clusters on Virus Containing Endosomes and Lysosomes Early in the Influenza A Infection of Human Airway Epithelial Cells. Viruses.

[13] Li K., Markosyan R.M., Zheng Y.M., Golfetto O., Bungart B., Li M., Ding S., He Y., Liang C., Lee J.C. (2013). IFITM Proteins Restrict Viral Membrane Hemifusion. PLoS Pathog..

[14] Klein S., Golani G., Lolicato F., Lahr C., Beyer D., Herrmann A., Wachsmuth-Melm M., Reddmann N., Brecht R., Hosseinzadeh M. (2023). IFITM3 blocks influenza virus entry by sorting lipids and stabilizing hemifusion. Cell Host Microbe.

[15] Clement M., Forbester J.L., Marsden M., Sabberwal P., Sommerville M.S., Wellington D., Dimonte S., Clare S., Harcourt K., Yin Z. (2022). IFITM3 restricts virus-induced inflammatory cytokine production by limiting Nogo-B mediated TLR responses. Nat. Commun..

[16] Xu J., Qian P., Wu Q., Liu S., Fan W., Zhang K., Wang R., Zhang H., Chen H., Li X. (2014). Swine interferon-induced transmembrane protein, sIFITM3, inhibits foot-and-mouth disease virus infection in vitro and in vivo. Antivir. Res..

[17] Kenney A.D., McMichael T.M., Imas A., Chesarino N.M., Zhang L., Dorn L.E., Wu Q., Alfaour O., Amari F., Chen M. (2019). IFITM3 protects the heart during influenza virus infection. Proc. Natl. Acad. Sci. USA.

[18] Mizushima N., Komatsu M. (2011). Autophagy: Renovation of cells and tissues. Cell.

[19] Taylor M.P., Kirkegaard K. (2007). Modification of Cellular Autophagy Protein LC3 by Poliovirus. J. Virol..

[20] Mizushima N., Yoshimori T. (2007). How to interpret LC3 immunoblotting. Autophagy.

[21] Wong J., Zhang J., Si X., Gao G., Mao I., McManus B.M., Luo H. (2008). Autophagosome supports coxsackievirus B3 replication in host cells. J. Virol..

[22] Zhou Z., Jiang X., Liu D., Fan Z., Hu X., Yan J., Wang M., Gao G.F. (2009). Autophagy is involved in influenza A virus replication. Autophagy.

[23] Lee Y.R., Lei H.Y., Liu M.T., Wang J.R., Chen S.H., Jiang-Shieh Y.F., Lin Y.S., Yeh T.M., Liu C.C., Liu H.S. (2008). Autophagic machinery activated by dengue virus enhances virus replication. Virology.

[24] Ait-Goughoulte M., Kanda T., Meyer K., Ryerse J.S., Ray R.B., Ray R. (2008). Hepatitis C Virus Genotype 1a Growth and Induction of Autophagy. J. Virol..

[25] Sun D., Kong N., Dong S., Chen X., Qin W., Wang H., Jiao Y., Zhai H., Li L., Gao F. (2022). 2AB protein of Senecavirus A antagonizes selective autophagy and type I interferon production by degrading LC3 and MARCHF8. Autophagy.

[26] Wen W., Li X., Yin M., Wang H., Qin L., Li H., Liu W., Zhao Z., Zhao Q., Chen H. (2021). Selective autophagy receptor SQSTM1/ p62 inhibits Seneca Valley virus replication by targeting viral VP1 and VP3. Autophagy.

[27] Hou L., Dong J., Zhu S., Yuan F., Wei L., Wang J., Quan R., Chu J., Wang D., Jiang H. (2019). Seneca valley virus activates autophagy through the PERK and ATF6 UPR pathways. Virology.

[28] Kudchodkar S.B., Levine B. (2009). Viruses and autophagy. Rev. Med. Virol..

[29] Choi Y., Bowman J.W., Jung J.U. (2018). Autophagy during viral infection—A double-edged sword. Nat. Rev. Microbiol..

[30] Jiang L.Q., Xia T., Hu Y.H., Sun M.S., Yan S., Lei C.Q., Shu H.B., Guo J.H., Liu Y. (2018). IFITM3 inhibits virus-triggered induction of type I interferon by mediating autophagosome-dependent degradation of IRF3. Cell. Mol. Immunol..

[31] Gu T., Yu D., Xu L., Yao Y.L., Yao Y.G. (2021). Tupaia GBP1 Interacts with STING to Initiate Autophagy and Restrict Herpes Simplex Virus Type 1 Infection. J. Immunol..

[32] Shi G., Ozog S., Torbett B.E., Compton A.A. (2018). mTOR inhibitors lower an intrinsic barrier to virus infection mediated by IFITM3. Proc. Natl. Acad. Sci. USA.

[33] Hales L.M., Knowles N.J., Reddy P.S., Xu L., Hay C., Hallenbeck P.L. (2008). Complete genome sequence analysis of Seneca Valley virus-001, a novel oncolytic picornavirus. J. Gen. Virol..

[34] Zhang X., Zhu Z., Yang F., Cao W., Tian H., Zhang K., Zheng H., Liu X. (2018). Review of seneca valley virus: A call for increased surveillance and research. Front. Microbiol..

[35] Segales J., Barcellos D., Alfieri A., Burrough E., Marthaler D. (2017). Senecavirus A: An emerging pathogen causing vesicular disease and mortality in pigs?. Vet. Pathol..

[36] Maggioli M.F., Fernandes M.H.V., Joshi L.R., Sharma B., Tweet M.M., Noll J.C.G., Bauermann F.V., Diel D.G. (2019). Persistent Infection and Transmission of Senecavirus A from Carrier Sows to Contact Piglets. J. Virol..

[37] Caserta L.C., Noll J.C.G., Singrey A., Niederwerder M.C., Dee S., Nelson E.A., Diel D.G. (2022). Stability of Senecavirus A in animal feed ingredients and infection following consumption of contaminated feed. Transbound. Emerg. Dis..

[38] Jia M., Sun M., Tang Y.D., Zhang Y.Y., Wang H., Cai X., Meng F. (2022). Senecavirus A Entry Into Host Cells Is Dependent on the Cholesterol-Mediated Endocytic Pathway. Front. Vet. Sci..

[39] Hou L., Tong X., Pan Y., Shi R., Liu C., Guo J., Shi Y., Yang X., Wang Y., Feng X. (2022). Seneca Valley Virus Enters PK-15 Cells via Caveolae-Mediated Endocytosis and Macropinocytosis Dependent on Low-pH, Dynamin, Rab5, and Rab7. J. Virol..

[40] Liu W., Shang X., Wen W., Ren X., Qin L., Li X., Qian P. (2023). Seneca Valley virus enters cells through multiple pathways and traffics intracellularly via the endolysosomal pathway. J. Gen. Virol..

[41] Anafu A.A., Bowen C.H., Chin C.R., Brass A.L., Holm G.H. (2013). Interferon-inducible transmembrane protein 3 (IFITM3) restricts reovirus cell entry. J. Biol. Chem..

[42] Pletan M.L., Tsai B. (2022). Non-enveloped virus membrane penetration: New advances leading to new insights. PLoS Pathog..

[43] Pang Z., Hao P., Qu Q., Li L., Jiang Y., Xiao S., Jin N., Li C. (2022). Interferon-Inducible Transmembrane Protein 3 (IFITM3) Restricts Rotavirus Infection. Viruses.

[44] Xu F., Dang W., Li T., Wang Y., Yang F., Zheng H. (2022). IFIT3 mediated the type I interferon antiviral response by targeting Senecavirus A entry, assembly and release pathways. Vet. Microbiol..

[45] Brass A.L., Huang I.C., Benita Y., John S.P., Krishnan M.N., Feeley E.M., Ryan B.J., Weyer J.L., van der Weyden L., Fikrig E. (2009). The IFITM proteins mediate cellular resistance to influenza A H1N1 virus, West Nile virus, and dengue virus. Cell.

[46] Joshi L.R., Fernandes M.H.V., Clement T., Lawson S., Pillatzki A., Resende T.P., Vannucci F.A., Kutish G.F., Nelson E.A., Diel D.G. (2016). Pathogenesis of Senecavirus A infection in finishing pigs. J. Gen. Virol..

[47] Spearman C. (1908). The method of right and wrong cases (constant stimuli) without Gauss’s formulae. Br. J. Psychol..

[48] Karber G. (1931). Beitrag zur kollektiven Behandlung pharmakologischer Reihenversuche. Arch. Exp. Path. Pharmacol..

[49] Livak K.J., Schmittgen T.D. (2001). Analysis of relative gene expression data using real-time quantitative PCR and the 2(-Delta Delta C(T)) Method. Methods.

[50] Steven Lawson D.D., Nelson E., Singrey A., Christopher-Hennings J. (2018). Development of A bELISA for Serological Diagnostics and Surveillance of SVA Infection.

[51] Schindelin J., Arganda-Carreras I., Frise E., Kaynig V., Longair M., Pietzsch T., Preibisch S., Rueden C., Saalfeld S., Schmid B. (2012). Fiji: An open-source platform for biological-image analysis. Nat. Methods.

[52] Yount J.S., Karssemeijer R.A., Hang H.C. (2012). S-palmitoylation and ubiquitination differentially regulate interferon-induced transmembrane protein 3 (IFITM3)-mediated resistance to influenza virus. J. Biol. Chem..

[53] Chesarino N.M., McMichael T.M., Yount J.S. (2015). E3 Ubiquitin Ligase NEDD4 Promotes Influenza Virus Infection by Decreasing Levels of the Antiviral Protein IFITM3. PLoS Pathog..

[54] Smith E.C., Popa A., Chang A., Masante C., Dutch R.E. (2009). Viral entry mechanisms: The increasing diversity of paramyxovirus entry. FEBS J..

[55] Tsai B. (2007). Penetration of nonenveloped viruses into the cytoplasm. Annu. Rev. Cell Dev. Biol..

[56] Sir D., Tian Y., Chen W.L., Ann D.K., Yen T.S., Ou J.H. (2010). The early autophagic pathway is activated by hepatitis B virus and required for viral DNA replication. Proc. Natl. Acad. Sci. USA.

[57] Berryman S., Brooks E., Burman A., Hawes P., Roberts R., Netherton C., Monaghan P., Whelband M., Cottam E., Elazar Z. (2012). Foot-and-mouth disease virus induces autophagosomes during cell entry via a class III phosphatidylinositol 3-kinase-independent pathway. J. Virol..

[58] O’Donnell V., Pacheco J.M., LaRocco M., Burrage T., Jackson W., Rodriguez L.L., Borca M.V., Baxt B. (2011). Foot-and-mouth disease virus utilizes an autophagic pathway during viral replication. Virology.

