# Supplementary figures and images for: Silencing RNA-Mediated Knockdown of IFITM3 Enhances Senecavirus A Replication
Source: Pathogens. 2024 Mar 29;13(4):290. doi: 10.3390/pathogens13040290 (PMC11054092; doi:10.3390/pathogens13040290)

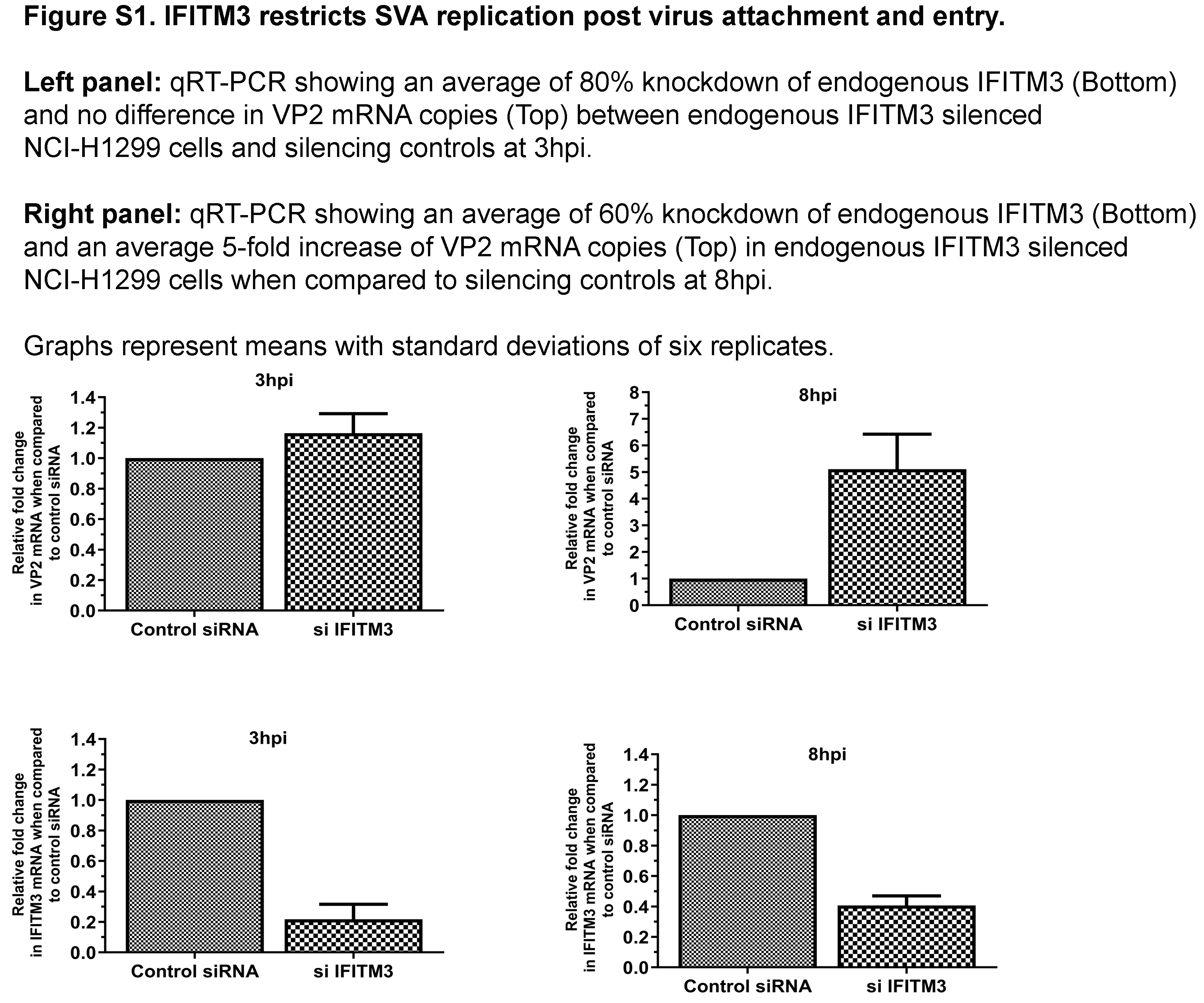

Supplement: Supplementary file 1 [file pathogens-13-00290-s001.zip › Supplementary Figure S1.tif]
